# The Effect of Light Intensity and the Timing of Light Exposure on Choroidal Thickness

**DOI:** 10.1007/s44402-026-00063-x

**Published:** 2026-03-25

**Authors:** Azam Darvishi, Scott A. Read, Stephen J. Vincent, David Alonso-Caneiro

**Affiliations:** 1 Contact Lens and Visual Optics Laboratory, Centre for Vision and Eye Research, Optometry and Vision Science, Queensland University of Technology, Brisbane, QLD Australia; 2 School of Science, Technology and Engineering, University of Sunshine Coast, Sunshine Coast, QLD Australia

**Keywords:** Choroidal thickness, Darkness, Light intensity, Myopia

## Abstract

**Purpose:**

To investigate the short-term effects of different light intensities on choroidal thickness and determine if these responses vary with time of day and refractive error.

**Methods:**

Twenty young adults (10 myopes, 10 emmetropes; mean age 29 ± 4 years; mean left-eye spherical equivalent −2.53 ± 1.84 and −0.12 ± 0.20 D, respectively, were exposed to three lighting conditions: bright light (2000 lx), normal room light (350 lx) and darkness (<0.1 lx) for 60 min in a randomised order on 6 separate days, in the morning (09:00–11:00 h) and evening (17:00–19:00 h), with at least 24 h between visits and all sessions completed within a 1-month period. Choroidal thickness of the left eye was measured at baseline, during 60 min of light exposure and 30 min post-exposure, to assess recovery.

**Results:**

Exposure to 2000 and <0.1 lx induced significant subfoveal choroidal thickening during the exposure period, peaking at 60 min (bright: +16 ± 4 μm; dark: +13 ± 6 μm; both *p* < 0.05), with values returning to baseline after 30 min of recovery. No significant changes occurred under 350 lx (*p* = 0.17). The greatest thickening was at the fovea (+16 ± 5 μm bright; +13 ± 6 μm dark) and parafovea (+13 ± 7 μm bright; +9 ± 6 μm dark), with smaller perifoveal changes. Bright light induced greater thickening in the evening than in the morning (*p* = 0.004), while darkness showed no time-of-day effect (*p* = 0.69). No significant refractive group differences were found (*p* = 0.90).

**Conclusions:**

One hour of bright light or darkness caused transient choroidal thickening in young adults. Changes were most prominent in the foveal region, and bright light produced stronger evening responses. These findings demonstrate that short-term choroidal thickness is dynamically modulated by both light intensity and time of day.

Key points

- One hour of exposure to bright light or darkness induces measurable, transient thickening of the human choroid.
- The magnitude of light-induced choroidal thickening was greatest in central foveal regions and with evening exposure, highlighting the importance of time-of-day effects.
- These findings provide new evidence that choroidal thickness is dynamically modulated by environmental light intensity, with potential relevance for ocular growth regulation.

## Introduction

The choroid, the vascular layer between the retina and sclera, supports vision by nourishing the outer retina, regulating eye temperature and pressure. It is thought to play a role in the mechanisms regulating eye growth and myopia development [1]. Alterations in choroidal thickness have been implicated in both myopic and hyperopic ocular growth, with evidence suggesting that choroidal changes can occur rapidly and may precede axial eye growth [2, 3]. This positions choroidal thickness as a potential early biomarker for refractive error development and ocular growth regulation. Consequently, examining short-term choroidal responses to visual environmental stimuli in the human eye may yield important insights into the underlying mechanisms of myopic eye growth [2, 4, 5].

Experimental studies in animal models have demonstrated that changes in choroidal thickness are closely linked to refractive development, with choroidal thickening typically associated with slowed axial elongation and hyperopic shifts, and choroidal thinning being associated with accelerated eye growth and myopic shifts [1, 6]. Consistent with these findings, longitudinal studies in children have shown that thinner choroids are associated with faster axial elongation and an increased risk of myopia onset [2, 7, 8]. In established myopia, the choroid is generally thinner and exhibits structural alterations, including reductions in both stromal and vascular components, highlighting the potential role of the choroid as both a mediator and biomarker of ocular growth [4, 9].

Research in humans and animals indicates that increased outdoor and bright light exposure may reduce the onset and progression of myopia by slowing axial eye growth, making environmental light a key factor in refractive development [10–12]. High-intensity illuminance inhibits the development of form-deprivation myopia in chicks [13], guinea pigs [14], mice [15], tree shrews [16] and rhesus monkeys [17]. Similarly, bright light (~15,000 lx) exposure reduces the progression of lens-induced myopia in chicks [18] and guinea pigs. [19] However, this effect has not been observed consistently in primates such as rhesus monkeys [20]. Lan et al. reported that exposing chicks to (~15,000 lx) daily for a 5-day period resulted in significant choroidal thickening (with some time delay in the choroidal response following initial exposure) compared to chicks reared under normal indoor lighting levels (500 lx), suggesting a potential role of the choroid in the effects of bright light upon refractive development [12].

Human studies, utilising objective quantification of light exposure using wearable sensors, have shown that myopic children receive less daily light exposure compared to their emmetropic peers [10, 21, 22]. Increased time outdoors has also been shown to lower the risk of developing myopia and slow axial eye growth in school-aged children [11, 23], with children exposed to more frequent, bright outdoor light (more than 1000 lx) experiencing reduced axial elongation of the eye [11, 24]. Read et al. reported that increased daily light exposure in Australian children aged 10–15 years was associated with slower eye growth [10], and similar findings in young adults show that greater time in bright light (>1000 lx) is linked to slower axial growth, with seasonal differences also being observed [25]. However, despite consistent associations reported in observational studies, the extent to which bright light exposure exerts a direct protective effect against myopia development in humans remains under debate [21].

Recent human studies have shown that short-term alterations in light exposure can induce changes in choroidal thickness, although responses vary depending on light characteristics and timing [3, 26–29]. Morning exposure to blue-green or narrowband cyan light has been associated with increased choroidal thickness and reduced axial length in both children and young adults [3, 26]. Similarly, short-term choroidal thickening has been reported following 2 h of morning exposure to bright broadband LED illuminance (1000 lx) compared with darkness (~5 lx) [27]. In contrast, night-time exposure to 1000 lx light over multiple nights has been associated with subfoveal choroidal thinning, suggesting a time-of-day dependence of light-induced choroidal changes [28]. High-intensity outdoor light exposure (≥1000 lx) has also been reported to induce choroidal thinning compared with indoor illumination (350 lx) and dark conditions (<0.1 lx) [29]. Darkness exposure may further modulate choroidal thickness in a time-dependent manner. While evening dark (0.0 cd/m^2^) adaptation has been associated with increased subfoveal choroidal thickness [30], other studies have reported choroidal thinning or no significant change following morning darkness exposure (*<*0.1–5 lx) [27, 29, 31].

To date, most studies examining light-related or circadian influences on the choroid have focused primarily on subfoveal choroidal thickness, given its high cone density, metabolic demand and widespread use in the literature [26–28, 30]. In the current study, choroidal thickness was assessed both subfoveally and across defined macular regions (foveal, parafoveal and perifoveal), which represents a novel aspect of the present study, enabling characterisation of the spatial distribution of choroidal modulation and providing additional insight into whether light-induced changes are localised or more broadly distributed across macular regions. Although several studies have explored the human choroidal response to altered light exposure [3, 21, 24, 27, 29, 31], inconsistent findings highlight the need for a clearer understanding of how different lighting conditions influence the choroid. While the outcomes from these studies suggest a potential influence of time of day on the choroidal response to altered light, no study has examined this systematically, particularly in the context of the well-established diurnal variation in choroidal thickness [32]. To understand better how altered light exposure influences the choroid and how these effects may interact with intrinsic diurnal rhythms, the current study investigated the effects of exposure to different light intensities (normal: 350 lx; bright: 2000 lx; darkness: <0.1 lx) upon choroidal thickness, exploring the time course of change and the influence of time of day (morning vs. evening) and refractive error in young adults.

## Methods

### Participants

Twenty healthy young adults (12 males and 8 females), aged 18–35 years, primarily from the student population at Queensland University of Technology (QUT), Brisbane, Australia, participated in this study. All participants had visual acuity of 0.00 logMAR or better in each eye and non-cycloplegic refractive errors of <6.00 D of myopia, <1.00 D of hyperopia, <1.00 D of astigmatism and <1.00 D of anisometropia.

Participants had no history of systemic or ocular diseases, eye injuries, binocular vision abnormalities, abnormal pupil responses and were not using any medications. None of the participants were undergoing myopia control treatment, such as orthokeratology, multifocal spectacles or contact lenses or atropine therapy and none wore rigid contact lenses. Binocular vision status was assessed using standard clinical tests, including the cover test and Howell heterophoria test. Ocular health was evaluated using slit-lamp biomicroscopy, and a detailed ocular and systemic health history was obtained from each participant. Spectral-domain optical coherence tomography (OCT) imaging was also performed during the initial screening session to assess posterior eye health. All of these criteria were assessed and confirmed during the initial screening session. To minimise potential issues with biometry [33] and visual performance [34] caused by contact lens use, participants were instructed to refrain from wearing contact lenses on the day of the experiment.

Participants were classified based on the refractive status of the left eye as either emmetropes (spherical equivalent refractive error (SER) between +0.75 DS and −0.50 DS) or myopes (SER between −0.75 DS and −6.00 DS), with an equal number of individuals in each category. Only left-eye data were analysed, reflecting the experimental optical setup (including the cold mirror and Badal lens system) configured for left-eye measurements (see Fig. S1). This approach minimised imaging time and avoided inter-eye dependency; therefore, refractive classification was based on the left eye. Since choroidal thickness changes in response to strenuous physical activity [35], caffeine [5] or alcohol consumption [36], participants were asked to avoid consuming caffeine or alcohol and refrain from strenuous exercise for at least 2 h before the experiment. Additionally, since cigarette smoking influences choroidal thickness [37, 38], smokers were also excluded from the study.

Participants were also screened to exclude those with conditions known to disrupt circadian rhythms, such as jet lag or insomnia. During the initial visit, participants completed the Pittsburgh Sleep Quality Index (PSQI) questionnaire to screen for sleep disorders [39]. Experimental study visits commenced within a maximum of 1 week following the initial screening visit. Approval from the QUT Human Research Ethics Committee was obtained prior to the study, all participants provided written informed consent and were treated in accordance with the principles of the Declaration of Helsinki.

### Procedures

**Fig. 1:**
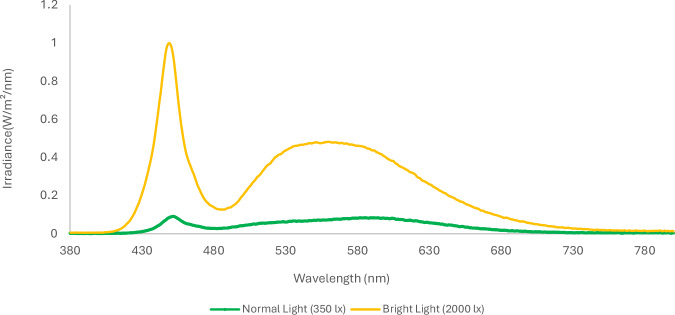
Spectral profiles of the normal (350 lx) and bright (2000 lx) lighting conditions used in the experiment, measured using a spectroradiometer. Spectral irradiance is expressed in watts per square metre per nanometre (W/m²/nm).

After the initial screening, six study sessions were conducted over 6 days, in a randomised order, to explore the impact of different illuminance intensities and the time of day on the choroid. Participants had choroidal imaging and axial length measurements captured before, during and after exposure to dark (<0.1 lx), normal (350 lx) and bright (2000 lx) ambient lighting conditions, with exposures occurring in both the morning and evening. The spectral characteristics of the normal (350 lx) and bright light (2000 lx) conditions were measured using a Stellar Net BLACK-Comet-CXR spectroradiometer (StellarNet Inc., stellarnet.us) (Fig. 1). During the exposure period for the normal and bright conditions, to encourage relaxed accommodation, participants viewed a grayscale movie presented on a wall-mounted projection screen located 4 m in front of the participant (screen luminance 5.5–6.5 cd/m²). For the dark condition, participants were seated 4 m from the screen (with no movie projected) and listened to a music podcast throughout the 60 min of darkness.

OCT and biometry measurements were performed in the same experimental room, required only a few minutes to complete, and did not interrupt the 60-min light or dark exposure period, which continued uninterrupted throughout each session. Participants were seated near the imaging devices and only needed to turn and position their head in the chin rest to allow OCT and biometry measurements to be acquired. All OCT and biometry measurements were performed under the same lighting conditions as the corresponding experimental exposure.

In the dark condition (<0.1 lx), all lights in the testing room were turned off, and any electronic devices in the lab were covered to maintain darkness (<0.1 lx). During the dark condition, participants remained in complete darkness (<0.1 lx) for the entire 60-min exposure period with all room lights switched off. Participants were seated immediately adjacent to the OCT and biometry instruments, allowing them to place their head directly into the chin rest without navigating through the laboratory in darkness. Eye alignment and focusing during OCT imaging were achieved using the instrument’s built-in near-infrared illumination, which is not visible to participants and does not alter ambient illuminance. OCT and biometry measurements were performed under black opaque cloths covering the OCT and Lenstar LS900 (Haag-Streit AG, haag-streit.com) devices, including their displays, to prevent any unintended light exposure.

Normal lighting (350 lx) was achieved with all ceiling-mounted LED laboratory room lights turned on, in combination with the screen luminance, to create an illuminance of 350 lx. For bright light conditions, an illuminance of 2000 lx was provided using two light stands placed 90 cm in front of the participant (in conjunction with the standard room lighting and screen luminance). Each light source was fitted with a rectangular diffuser (20 × 30 cm), which at this viewing distance subtended a visual angle of ~18° horizontally and 13° vertically. These light stands were positioned symmetrically on either side of the participant’s midline, separated by ~30 cm (each stand was 9° from the line of sight), at eye level. The light stands were angled slightly inward towards the participant to ensure overlapping illumination fields. During exposure, participants directed their fixation between the two light stands to view the projected movie on the screen positioned behind the stands.

Each light stand consisted of an Epistar 48-W LED spotlight (Ennostar Corporation, chip.ennostar.com) with a colour temperature of 6000 K combined with a 20 cm × 30 cm Lee Quarter White (251) diffusing filter (LEE Filters, leefilters.com), to reduce glare from the LED sources and make the condition more comfortable for participants. The spatial arrangement and orientation of the light stands relative to the participant’s eyes are illustrated in Supplementary Fig. S2. These light stands remained in the same position in the laboratory for all conditions, but were only switched on for the 2000 lx condition. This configuration ensured that bright-light conditions produced elevated illumination across the central 40° macula region, with a peak in illumination in the central foveal region (due to the overlapping fields of the diffuse light sources). Illuminance was measured using a lux metre positioned at the participant’s eye level, aligned with the direction of gaze.

Table 1 presents the lighting characteristics and the α-opic irradiance values for individual photoreceptor excitation under the various lighting conditions, calculated using the CIE S 026 alpha-opic Toolbox [40].

**Table 1:** Spectral characteristics and individual photoreceptor excitation (alpha-opic irradiance) for the bright (2000 lx) and normal (350 lx) light conditions.  lx lux.

Condition	Total irradiance (W/m^2^)	Illuminance (lx)	Photoreceptor excitation (alpha-opic W/m^2^)				
			S cone	M cone	L cone	Rod	Melanopsin
Bright light (2000 lx)	6.74	2122.61	1.69	2.91	3.40	2.48	2.12
Normal light (350 lx)	1.08	357.44	0.16	0.46	0.58	0.36	0.30

Irradiance is expressed in watts per square metre (W/m²). S-, M- and L-cones denote short-, medium- and long-wavelength–sensitive cone photoreceptors, respectively.

Before each session, participants underwent a 20-minute wash-out period to relax accommodation and reduce the potential effects of prior visual tasks on the measurements by watching a grayscale movie at 4-metres under dim (10 lux) illuminance. This target ambient illumination of 10 lux at eye level was achieved by the projected movie, in combination with dim indoor room lighting. Measurements were taken before (0 min), during exposure (at time points 10, 20, 30 and 60 min exposure) and after a 30-min recovery period under standard room illuminance (350 lx). The morning sessions were scheduled between 09:00 and 11:00 h and the evening sessions between 17:00 and 19:00 h. Each participant completed their measurements within a month, with at least 24 h between each session. During the 1-h exposure to different light intensities (normal lighting at 350 lx, bright lighting at 2000 lx) and during the 30-min recovery period, participants watched a movie projected on a screen 4 m away (Table 2).

**Table 2:** Overview of the experimental protocol. lx lux, OCT optical coherence tomography.

	Before exposure	During exposure				After exposure
Lighting	20-min wash-out (10 lx)	Dark (<0.1 lx)				30-min recovery (350 lx)
		Normal light (350 lx)				
		Bright light (2000 lx)				
Timing (min)	0	10	20	30	60	90
Measurement (morning/evening)	OCT	OCT	OCT	OCT	OCT	OCT
	Biometry				Biometry	Biometry

Participants completed six experimental sessions on separate days in a randomised order to assess choroidal thickness and axial length before, during and after exposure to various lighting conditions, both in the morning and evening.

The Heidelberg Spectralis optical coherence tomographer (OCT) (Spectralis SD-OCT, Heidelberg Engineering, heidelbergengineering.com) was used to assess choroidal thickness. This instrument is a spectral domain-OCT with a scanning laser ophthalmoscope to allow eye tracking and provides high-resolution cross-sectional chorioretinal images. During each OCT measurement taken at 0, 10, 20, 30, 60 and 90 min, the left eye focused on an external fixation target—a red cross displayed on a Samsung Galaxy S7 smartphone (Samsung Electronics, samsung.com) positioned at the centre of an LCD screen, with a luminance of 71 cd/m². This target was viewed at optical infinity through a +15 D Badal lens and a cold mirror attached to the objective lens of the OCT, while the internal bright blue fixation target of the instrument remained switched off to ensure that exposure to this target did not confound the results.

OCT imaging was repeated three times at each time point on the left eye. using the first image captured at 0 min as a reference, to ensure that all follow-up scans were taken from the same retinal location using the OCT system’s built-in eye-tracking function. All scans were inspected to confirm foveal centration, including the presence of the foveal pit. The external fixation setup is illustrated in Fig. S1. The OCT imaging was performed in enhanced depth imaging mode with a foveal-centred 30° horizontal line scan. Each image was generated by averaging 100 repeated B-scans (automatic real-time averaging) to improve image quality and the signal-to-noise ratio. The scans were acquired with an axial resolution of 3.9 μm. The intraobserver repeatability for choroidal thickness at the macula for this instrument has been reported to fall within a range of ±13 μm [41, 42]. Ocular biometry was captured using the Lenstar LS900 instrument (Haag-Streit AG, haag-streit.com) at baseline, after 60 min of exposure and 30 min of recovery. This instrument utilises optical low coherence reflectometry and provides precise measurements of axial length [43].

### Data Analysis

#### Choroidal Thickness

**Fig. 2:**
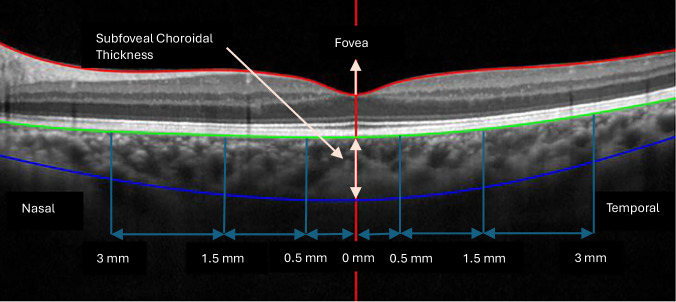
A representative chorioretinal optical coherence tomography (OCT) scan, obtained from a healthy young adult. Segmentation of the retinal pigment epithelium (RPE) (green line) and chorioscleral interface (blue line) enabled the measurement of choroidal thickness. The blue double-headed arrows beneath the scan indicate the different zones quantified, including the foveal zone (from the foveal centre to 0.5 mm), the parafoveal zone (0.5–1.5 mm from the foveal centre), and the perifoveal zone (1.5–3.0 mm from the foveal centre).

The OCT images were exported from the instrument and analysed using custom-developed software in a semi-automated process [44]. For each image, the outer border of the retinal pigment epithelium (RPE) and the inner border of the chorioscleral interface were automatically segmented across each scan. An experienced observer, masked to the experimental conditions and refractive group of the participants, then reviewed the integrity of the automated segmentation and manually corrected any segmentation errors. Additionally, the observer identified the central foveal point manually, representing the deepest point of the foveal pit (Fig. 2).

The subfoveal choroidal thickness was determined by measuring the axial distance between the outer boundary of the RPE and the inner boundary of the chorioscleral interface at the foveal centre. The average choroidal thickness was also determined within different zones surrounding the fovea, within the central 6 mm of each scan. These zones comprised: (i) the foveal zone: from the foveal centre to 0.5 mm away, (ii) the parafoveal zone: from 0.5 to 1.5 mm from the foveal centre and (iii) the perifoveal zone: from 1.5 to 3 mm from the foveal centre [2, 45]. Since axial length can influence the lateral scale of OCT images, individual differences in axial length were accounted for by recalibrating the transverse scan dimensions based upon each participant’s measured axial length at the corresponding time point (baseline, 60 and 90 min), following an approach similar to Wagner-Schuman et al. [46].

### Statistical Analysis

The choroidal thickness data were analysed using IBM SPSS version 29 (ibm.com). A repeated-measures analysis of variance (ANOVA) with a split-plot (mixed-design) structure was used to assess the within-subject effects of lighting condition, exposure duration, time of measurement and time of day on changes in choroidal thickness. A between-subjects factor of refractive group was also included to examine differences between myopes and emmetropes. The regional differences in choroidal metrics were analysed using an additional repeated measures ANOVA, incorporating lighting condition, time of measurement, time of day and refractive error group, as well as choroidal eccentricity (the foveal, parafoveal and perifoveal zones) and choroidal meridian (nasal and temporal), along with all possible interactions. In these analyses, lighting condition, time of measurement, time of day, eccentricity and meridian were treated as within-subject factors, while refractive group was treated as a between-subject factor.

Bonferroni-adjusted pairwise comparisons were used to explore any significant main effects and interactions. *P*-values < 0.05 were considered statistically significant. Data from pilot studies indicated that a sample size of 20 participants would provide 90% power to detect a 6 µm change in choroidal thickness between different light intensities at the 0.05 significance level.

### Ethics approval

Ethics approval was granted by the QUT Human Research Ethics Committee (approval number: 7836).

## Results

### Participant Characteristics

Demographic and refractive characteristics of the 20 healthy participants (mean ± SD age: 29 ± 4 years) are summarised in Table 3. The mean SER of the left eyes was −1.33 ± 1.7 D (range: −5.75 to +0.25 D). The cohort included both emmetropic and myopic participants, with no significant differences in age (*p* = 0.44) or baseline choroidal thickness between groups (*p* = 0.08), while, as expected, SER error differed significantly (*p* = 0.003).

**Table 3:** Baseline characteristics of the left eye are shown as mean ± standard deviation (range) unless otherwise indicated.

Value	Myopes	Emmetropes	*P*
*N* (male)	10(6)	10(6)	
Age (years)	28 ± 4 (21–35)	29 ± 4 (22–34)	0.44
Spherical equivalent (D)	−2.53 ± 1.84 (−0.75 to −5.75)	−0.12 ± 0.25 (+0.25 to −0.50)	0.003
Subfoveal choroidal thickness (µm)	313 ± 64 (194–414)	370 ± 72 (262–467)	0.08

*P*-values are from an independent samples *t*-test comparing myopes and emmetropes. Subfoveal choroidal thickness values reflect the mean of baseline (0 min) measurements collected across six study sessions.

### Baseline Subfoveal Choroidal Thickness

Baseline subfoveal choroidal thickness measurements were obtained at the start of each experimental session (0 min), prior to light exposure (Table 4). Mean baseline choroidal thickness values were marginally greater during the evening sessions compared with the morning sessions across all lighting conditions. However, pairwise comparisons derived from the repeated-measures ANOVA demonstrated no statistically significant differences in baseline subfoveal choroidal thickness between morning and evening sessions for 2000 lx (*p* = 0.28), <0.1 lx (*p* = 0.91) or 350 lx conditions (*p* = 0.08).

**Table 4:** Baseline subfoveal choroidal thickness (ChT; mean ± standard deviation) measured in the morning and evening sessions.

	Bright light (2000 lx)	Darkness (<0.1 lx)	Normal light (350 lx)
Subfoveal ChT-Morning (µm)	340 ± 73	342 ± 73	341 ± 73
Subfoveal ChT-Evening (µm)	342 ± 71	342 ± 72	344 ± 21

### Subfoveal Choroidal Thickness

**Fig. 3:**
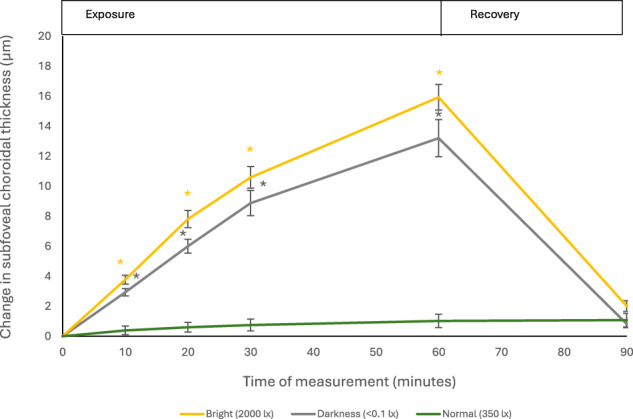
The mean change in subfoveal choroidal thickness in response to 2000 lx (yellow line), <0.1 lx (grey line) and 350 lx (green line), averaged across the morning and evening testing sessions. Error bars represent the standard error of the mean. Yellow and grey asterisks signify significant (*p* < 0.05) choroidal changes in bright light (2000 lx) and darkness (<0.1 lx) compared to the baseline (0 min) measurement, respectively.

Repeated measures ANOVA revealed significant main effects of lighting condition (*F*(2,36) = 103.15, *p* < 0.001, partial *η*² = 0.851), time of measurement (*F*(5,90) = 213.43, *p* < 0.001, partial *η*² = 0.922) and a lighting by time of measurement interaction (*F*(10,180) = 70.26, *p* < 0.001, partial *η*² = 0.796). A significant progressive thickening of the choroid was observed over the 60 min exposure period in response to 2000 and <0.1 lx, but no significant changes were found in response to 350 lx (Fig. 3).

Bonferroni adjusted pairwise comparisons revealed that a significant thickening of the choroid, compared to baseline, was evident at 10 min (mean change +4 ± 1 μm), 20 min (+8 ± 3 μm), 30 min (+11 ± 3 μm) and 60 min (+16 ± 4 μm) after exposure to 2000 lx (all *p* < 0.001). Similarly, significant choroidal thickening was noted following exposure to <0.1 lx at 10 minutes (mean change +3 ± 1 μm), 20 min (+6 ± 2 μm), 30 min (+9 ± 4 μm) and 60 min (+13 ± 6 μm) (all *p* < 0.001). While the average increase in choroidal thickness was greater with 2000 lx exposure than for <0.1 lx, the differences observed at each time point were not statistically significant (all *p* > 0.05). For both the bright light (2000 lx) and darkness (<0.1 lx) conditions, on average, the choroidal thickness changes had returned to baseline levels following 30 min in 350 lx. There were no significant changes in choroidal thickness from baseline at any time points during the normal light conditions (350 lx) (all *p* > 0.05).

**Fig. 4:**
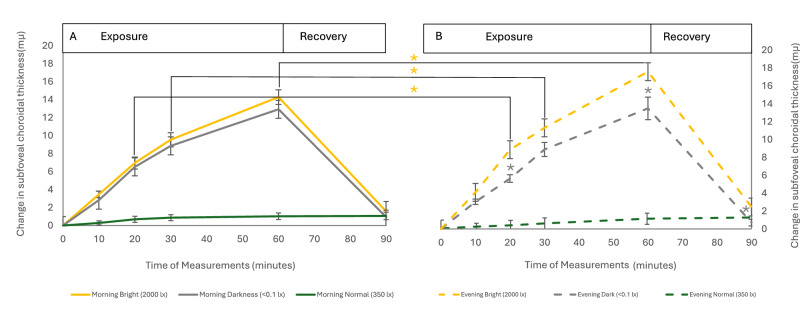
The mean change in subfoveal choroidal thickness during the morning (09:00–11:00 h; solid lines) (**A**) and the evening (17:00–19:00 h; dashed lines) (**B**). Green, grey and yellow lines represent 350, <0.1 and 2000 lx, respectively. Yellow asterisks signify a significant difference (*p* < 0.05) in choroidal changes between the morning and evening for bright light (2000 lx). Grey asterisks indicate a significant difference in choroidal changes between bright light (2000 lx) and darkness (<0.1 lx) (*p* < 0.05). Error bars represent the standard error of the mean.

A significant time of day by lighting condition by time of measurement interaction (*F*(10,180) = 3.92, *p* = 0.004, partial *η*² = 0.179) and a significant time of day by lighting condition interaction (*F*(2,36) = 7.18, *p* = 0.004, partial *η*² = 0.285) was observed, indicative of differences in the changes over time in response to some lighting conditions between the morning and evening measurement sessions. For both the darkness (<0.1 lx) and bright light (2000 lx) exposure conditions, pairwise comparisons showed a significant thickening of the choroid following 10, 20, 30 and 60 min of exposure in both the morning and the evening measurement sessions (all *p* < 0.05) (Fig. 4). Choroidal thickness in the normal light condition (350 lx) showed no significant changes over time in either the morning or the evening measurement sessions (all pairwise comparisons *p* > 0.05).

For exposure to 2000 lx, the magnitude of choroidal thickening at 20, 30 and 60 min was significantly greater in the evening compared to the morning (mean differences of +2 ± 3 μm at 20 and 30 min, *p* = 0.005 and *p* = 0.007, respectively and +3 ± 4 μm at 60 min, *p* = 0.01). In the morning, the magnitude of choroidal thickening for the darkness (<0.1 lx) and bright light (2000 lx) exposure conditions was not significantly different from each other (all comparisons *p* > 0.05). However, in the evening, a significantly greater thickening of the choroid was observed with bright light exposure compared to darkness (<0.1 lx) at 20 min (mean difference of 3 ± 4 μm), 60 min (mean difference of 4 ± 3 μm) and 90 min (mean difference of 2 ± 2 μm; all *p* < 0.05).

There was no significant main effect of refractive error (*F*(1,18) = 0.70, *p* = 0.50, partial *η*² = 0.04) or refractive error interactions (all *p* > 0.05) in relation to light exposure on choroidal thickness, indicating a similar response in the myopic and emmetropic participants.

### Regional Choroidal Responses

Analysis of mean regional choroidal thickness revealed significant main effects of eccentricity (*F*(2,36) = 22.64, *p* < 0.001, partial *η*² = 0.557), lighting condition (*F*(2,36) = 3.99, *p* = 0.03, partial *η*² = 0.181), meridian (*F*(1,18) = 12.87, *p* = 0.002, partial *η*² = 0.417) and time of measurement (*F*(5,90) = 46.06, *p* < 0.001, partial *η*² = 0.719).

There were statistically significant regional variations in mean choroidal thickness across both eccentricity (*F*(2,36) = 22.64, *p* < 0.001, partial *η*² = 0.557) and meridian (*F*(1,18) = 12.87, *p* = 0.002, partial *η*² = 0.417). Mean choroidal thickness was greatest in the foveal region (340 ± 72 μm), decreased in the parafovea (333 ± 71 μm) and was lowest in the perifovea (304 ± 68 μm), with all pairwise comparisons reaching statistical significance (*p* < 0.05). In addition, mean choroidal thickness was significantly greater in the temporal meridian (340 ± 70 μm) compared to the nasal meridian (312 ± 72 μm) (*p* = 0.002).

**Fig. 5:**
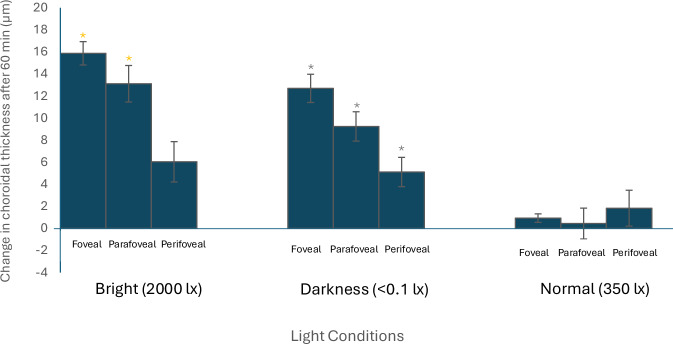
The mean choroidal thickness change in the foveal, parafoveal and perifoveal zones after 60 min exposure to 2000, <0.1 and 350 lx, averaged across the morning and evening testing sessions. Error bars represent the standard error of the mean. Asterisks signify a significant (*p* < 0.05) choroidal thickness change after 60 min compared to the baseline (0 min) measurement.

Consistent with the change in the subfoveal choroid, a significant progressive thickening of the choroid over the 60-min exposure period was found in response to the <0.1 and 2000 lx conditions. Significant interactions between eccentricity and lighting condition (*F*(4,72) = 2.61, *p* = 0.04, partial *η*² = 0.12), eccentricity by lighting condition by time of measurement (*F*(20,360) = 3.93, *p* < 0.001, partial *η*² = 0.17) and eccentricity by time of measurement (*F*(10,180) = 7.91, *p* < 0.001, partial *η*² = 0.305) were also observed. Figure 5 illustrates the choroidal changes after 60 min for each of the lighting conditions in the fovea, parafovea and perifovea. Under both <0.1 and 2000 lx, there was a greater change in thickness observed in the fovea, followed by the parafovea and then the perifovea. There was no significant meridian by lighting condition or meridian by lighting by time interactions (all *p* > 0.05).

Changes in choroidal thickness were significantly greater in the foveal and parafoveal regions compared to the perifoveal region. Bonferroni-adjusted pairwise comparisons revealed a significant increase in choroidal thickness at 60 min compared to baseline in the foveal (+16 ± 5 μm) and parafoveal (mean change +13 ± 7 μm) regions following 2000 lx exposure (all *p* < 0.001), whereas changes in the perifoveal region (mean change +6 ± 8 μm) were not significant (*p* = 0.42). Similarly, after exposure to <0.1 lx, significant choroidal thickening was observed at 60 min in the foveal (mean change +13 ± 6 μm), parafoveal (mean change +9 ± 6 μm) and perifoveal (mean change +5 ± 6 μm) regions (*p* < 0.05).

The choroidal changes at 60 min varied significantly with eccentricity. In response to 2000 lx, all comparisons were significant: fovea vs. perifovea (mean difference +10 ± 8μm, *p* < 0.001), parafovea vs. perifovea (mean difference +7 ± 5 μm, *p* < 0.001) and fovea vs. parafovea (+4 ± 4 μm, *p* < 0.001). Similarly, for the dark condition (<0.1 lx), significant changes were observed with eccentricity: fovea vs. perifovea (mean difference +8 ± 7 μm, *p* < 0.001), parafovea vs. perifovea (mean difference +4 ± 5 μm, *p* = 0.006) and fovea vs. parafovea (mean difference +4 ± 5 μm, *p* = 0.009). For 350 lx illuminance, no significant changes were found in any of the choroidal zones (all *p* > 0.05).

## Discussion

This study demonstrated that 60 min of exposure to both high-intensity light (2000 lx) and darkness (<0.1 lx) in young adults led to a significant increase in choroidal thickness. The choroidal response to 2000 and <0.1 lx was most prominent in the fovea, with smaller changes observed in the perifovea. The response to 2000 lx was also influenced by time of day, with a greater magnitude of thickening observed in the evening. The observed ocular changes were transient, with values reverting to baseline levels 30 min after exposure.

### Effects of Light Intensity on the Choroid

A limited number of studies have examined the effects of light intensity on choroidal thickness in humans, with indoor lighting conditions typically at or below 1000 lx [27] compared to 2000 lx in the current study. Using a similar methodology to the current study (LED light sources with comparable spectral power distributions that peaked around 450 nm), Chakraborty et al. [27] examined the choroidal response following 120 min of exposure to 500 and 1000 lx of white light. Comparing the results of Chakraborty et al. [27] with the current study suggests a dose-dependent effect of choroidal thickening with increasing broadband light intensities: +1 μm with 350 lx (108 μW/cm²), +6 μm with 500 lx (152 μW/cm²), +13 μm with 1000 lx (24 μW/cm²) and +16 μm with 2000 lx (674 μW/cm²).

Lou and Ostrin [29] also reported a negligible (~1 µm) reduction in choroidal thickness following 60 min of exposure to broadband white light at 350 lx, which is comparable in magnitude to the minimal change (~1 µm) observed under the 350 lx (108 µW/cm²) broadband normal lighting condition in the current study. However, exposure to intense natural sunlight in an outdoor environment induced choroidal thinning, a result that differs from the choroidal thickening observed in both the current study and that of Chakraborty et al. [27]. Differences in findings across studies may be explained by variations in their experimental designs. For example, Lou and Ostrin [29] exposed participants to natural sunlight, where light intensity frequently surpassed 2000 lx (6000–50,000 lx), compared to the precisely regulated artificial lighting conditions in laboratory studies. Other factors, such as differences in climate, temperature and spatial frequency content in different environments, may contribute to differences between studies [27]. Moreover, outdoor light exposure differs, not only in intensity but also in spectral composition, compared to indoor artificial lighting, including the lighting conditions used in the present study. Besides intensity, the spectral composition of light may play a role in ocular responses [3, 26]. The light source used in this study produced a broad increase in irradiance across the visible spectrum, with substantial increases in excitation for all photoreceptor classes under 2000 lx, compared to 350 lx (Table 1). Given this relatively broad increase, it is difficult to attribute the choroidal changes observed to a specific spectral range of exposure. However, as highlighted in Fig. 1, the relative magnitude of increase in irradiance for 2000 lx was greatest for shorter compared with longer wavelengths, including a pronounced peak in the blue region around 450 nm, and previous studies have reported choroidal thickening in response to narrowband short-wavelength light [27, 47]. This suggests that short-wavelength components of the bright light spectrum may have contributed to the observed choroidal responses, although wavelength-specific effects cannot be isolated in the present study.

### Regional Characteristics of Choroidal Responses

The choroidal response to light also showed regional variations, with the highest magnitude of thickening occurring at the fovea and smaller changes in more peripheral regions. This spatial pattern aligns with previous findings [47], where greater choroidal thickening was observed in the foveal region compared to more peripheral regions in response to blue light exposure, supporting the notion that the fovea is more sensitive to light-induced choroidal changes than peripheral regions. This greater central reactivity may reflect the higher metabolic demand and functional importance of the foveal region, suggesting that light-induced choroidal modulation may be most relevant in retinal areas with the greatest visual processing load [1, 4]. This spatial pattern may also have implications for understanding how environmental light influences ocular growth signals originating from the central retina, although further long-term studies are required to determine whether these short-term regional changes translate into sustained effects on eye growth [2, 47].

When interpreting the regional choroidal responses to blue light exposure, it is important to consider the non-uniform retinal distribution of light-sensitive retinal pathways with varying eccentricity. In particular, short-wavelength-sensitive (S) cones are absent or extremely sparse at the foveal centre, with their density increasing along the foveal slope and peaking in the parafoveal retina [48]. Similarly, melanopsin-expressing intrinsically photosensitive retinal ganglion cells (ipRGCs) are also largely absent from the fovea and show greater densities in the peri-/parafoveal retina [49, 50]. Together, these cone-mediated and melanopsin-mediated pathways may contribute to the spatial pattern of choroidal responses observed across retinal eccentricities. Furthermore, regional differences within the choroid itself, including regional variations in choroidal neurons and the distribution of non-vascular smooth muscle, may also contribute to the spatial patterns of observed choroidal responses [51, 52].

### Effects of Time of Day on the Choroid

Consistent with findings from studies in the chick model, the choroidal response to bright light (2000 lx) was also of greater magnitude in the evening compared to the morning. Sarfare et al. [53], showed that brief daily bright light exposure (30K lx) in the evening led to a greater increase in choroidal thickness compared to morning exposure in chicks. While the current study provides the first systematic examination of the effects of time of day on the choroidal response to light in the human eye, the findings are consistent with recent human research demonstrating greater choroidal thickening in the evening in response to myopic defocus, highlighting the importance of considering time-of-day effects in ocular growth studies [54]. In addition to potential differences in retinal sensitivity, the greater choroidal response observed in the evening may also reflect diurnal variation in choroidal responsiveness itself. Time-of-day-dependent changes in retinal and choroidal signalling pathways (e.g., dopamine and nitric oxide), autonomic regulation of choroidal blood flow and vascular reactivity may enhance the magnitude of light-induced choroidal thickening in the evening [10, 55].

Previous studies of diurnal variations in choroidal thickness suggest that over a short time interval of 60 min (the exposure period of the current study), any diurnal variations are expected to be minimal, typically only a few micrometres [32, 55]. To minimise the influence of such short-term diurnal fluctuations when examining light effects, the changes from session-specific baseline measurements were examined. Under the normal light condition (350 lx), which provided a comparison condition in the absence of strong light modulation, neither mean choroidal thickness nor baseline-normalised changes differed significantly between the morning and evening sessions, and no systematic changes were observed over the 60-min exposure period, suggesting minimal diurnal variation over the light exposure periods.

Although choroidal thickening occurred under both darkness and bright light exposure, the significantly greater magnitude of baseline-normalised thickening under bright light—particularly during the evening session—suggests an additional light-driven modulation superimposed on the underlying diurnal rhythm, rather than an effect attributable solely to time-of-day-related changes in mean choroidal thickness [2, 53, 55]. However, it should be noted that the magnitude of the evening–morning differences observed under bright light was relatively small, and should be interpreted with caution when considered in the context of the axial resolution of the OCT system (3.9 µm).

### Effects of Darkness on the Choroid

Interestingly, a significant thickening of the choroid was also observed after 1 h of exposure to <0.1 lx, which returned to baseline within 30 min of exposure to 350 lx. This finding is consistent with Alagöz et al. [30], who reported choroidal thickening after 30 min of exposure to <0.1 lx during the evening, but in contrast to other studies, which found that darkness caused no change [29] or choroidal thinning [27, 31]. Differences in the duration of exposure to <0.1 lx may partly explain variations in the magnitude and pattern of choroidal thickening reported across studies (e.g., 2 h exposure) [27, 29]. In addition to variations in exposure duration, some studies used different levels of dim light (e.g., 5 lx instead of complete darkness (<0.1 lx), which could further influence the extent of the choroidal response, with complete darkness potentially eliciting a stronger thickening effect. Additionally, in the current study, the instrument’s internal fixation light was turned off during measurements to reduce any confounding effect of instrument-generated illumination.

While the choroidal thickening observed during exposure to <0.1 lx may be a physiological response related to outer retinal metabolic demands [56], it could potentially have some implications for eye growth. The choroidal thickening in both <0.1 and 2000 lx aligns with findings reporting that myopic children experience significantly less exposure to both bright outdoor light (>1000 lx) and scotopic (<1 lx) compared to non-myopic children [57]. Furthermore, the previously reported correlation between increased time spent in mesopic light levels and greater myopic refractive error suggests that not only bright light but also dim light exposure may play a role in myopia development [57]. These insights underscore the importance of considering a broad range of ambient light conditions when exploring environmental factors influencing myopia. Another possible factor that could influence choroidal thickness in darkness is tonic accommodation. Although tonic accommodation may occur in darkness [58], its magnitude is typically small and unlikely to induce substantial choroidal thickness changes, as significant accommodation-related choroidal responses have generally been reported only for accommodative demands of ~ 3D or greater [59].

In comparing the effects of bright light and darkness (2000 and <0.1 lx), both conditions elicited a significant progressive thickening of the choroid over the 60-min exposure period; however, some differences in the magnitude and temporal pattern of change were observed, particularly in relation to time of day. While there were no statistically significant differences between the two lighting conditions in the morning, 2000 lx exposure in the evening led to significantly greater choroidal thickening than darkness (<0.1 lx) at 20, 60 and 90 min post-exposure. Furthermore, the thickening response to 2000 lx was significantly greater in the evening compared to the morning at 20 and 60 min, but no time-of-day effects were observed in response to <0.1 lx. These findings suggest that different mechanisms may underlie the choroidal thickening observed in response to <0.1 and 2000 lx, with the differences in the magnitude and time course of the response suggesting that distinct physiological pathways may be involved. Previous evidence indicates that the effects of 2000 lx may be mediated by increased retinal dopamine release [12, 27], which can also lead to choroidal thickening [12, 60]. It has been hypothesised that light-induced choroidal thickening is mediated by retinal dopamine and subsequent downstream effects such as nitric oxide release that affect the choroid [12]. On the other hand, the choroidal response to <0.1 lx may be linked to heightened metabolic activity in photoreceptors [61, 62], which increases oxygen consumption in the outer retina. While oxygen levels decline in the outer retina during darkness (<0.1 lx), oxygen tension within the choroid remains stable [63, 64]. This mismatch between demand and supply may lead to a compensatory thickening of the choroid to enhance oxygen delivery to the photoreceptors.

Read et al. [65] investigated the effects of short-term optical blur and demonstrated similar choroidal responses between adult myopes and emmetropes, which is consistent with the present findings of no significant difference between the two groups. This suggests that the mechanisms underlying choroidal adaptation to visual stimuli may operate similarly across refractive groups. Although both blur and illuminance can induce choroidal changes, the underlying mechanisms are likely distinct, and direct comparisons between these effects should be made cautiously.

Future research is warranted to elucidate further how specific wavelength composition, light intensity, exposure duration and darkness interact to influence choroidal thickness and their potential relevance to eye growth and myopia development. A limitation of the current study is the small sample size, composed solely of young adults, which restricts the applicability of the findings across different age groups. Moreover, since choroidal thickness was only assessed under bright light (2000 lx), darkness (<0.1 lx) and standard lighting (350 lx) during a limited 90-min timeframe, the potential dose–response effects related to exposure duration were not evaluated. Future investigations should consider larger, more heterogeneous populations and extended exposure periods to elucidate better the complex relationship between light conditions, timing and ocular structural changes.

In conclusion, exposure to bright light (2000 lx) and darkness (<0.1 lx) for 1 h led to significant choroidal thickening in young adults, while no notable changes were observed under normal lighting (350 lx). These alterations returned to baseline within 30 min of recovery. Additionally, a greater choroidal response was observed with 2000 lx exposure in the evening, but there were no significant differences between refractive groups. These findings highlight the dynamic and time-of-day-dependent nature of choroidal responses to light and darkness, advancing our understanding of physiological processes that may contribute to ocular growth regulation.

## Supplementary information

Supplementary information

## Data Availability

The data that support the findings of this study are available from the corresponding author upon reasonable request.
