# Supplementary material for: The Effect of Light Intensity and the Timing of Light Exposure on Choroidal Thickness
Source: Ophthalmic Physiol Opt. 2026 Mar 25;46(3):616–25. doi: 10.1007/s44402-026-00063-x (PMC13369561; doi:10.1007/s44402-026-00063-x)
Supplement: Supplementary file 1 — Supplementary information [file 44402_2026_63_MOESM1_ESM.pdf]

Title: **The effect of light intensity and the timing of light exposure on choroidal thickness**

Journal Ophthalmic and Physiological Optics
Authors: Azam Darvishi, Scott A Read, Stephen J Vincent , David Alonso-Caneiro

Corresponding author:
Azam Darvishi, Centre for Vision and Eye Research, Contact Lens and Visual Optics Laboratory, Queensland University of Technology, Brisbane, Queensland, Australia.

Email: azam.darvishi@hdr.qut.edu.au

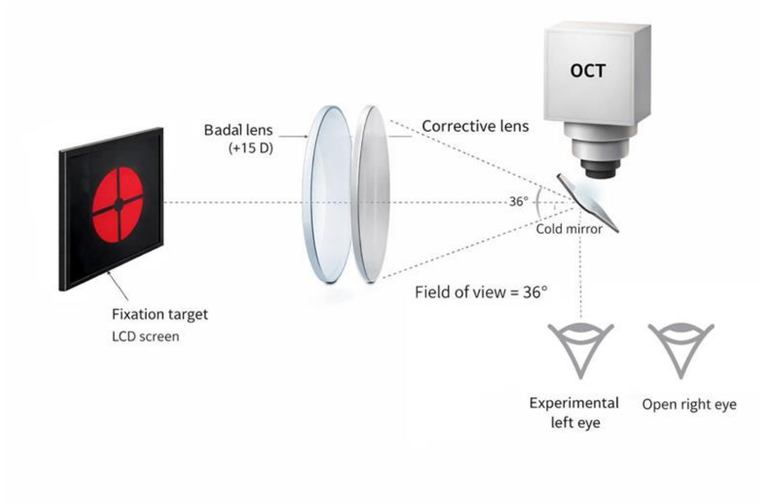

Supplementary Figure S1. Experimental OCT setup illustrating the use of an external fixation target presented on an LCD screen. The external target was viewed via the OCT optical pathway using a cold mirror (beam splitter), with a +15 D trial lens incorporated to achieve optical infinity.

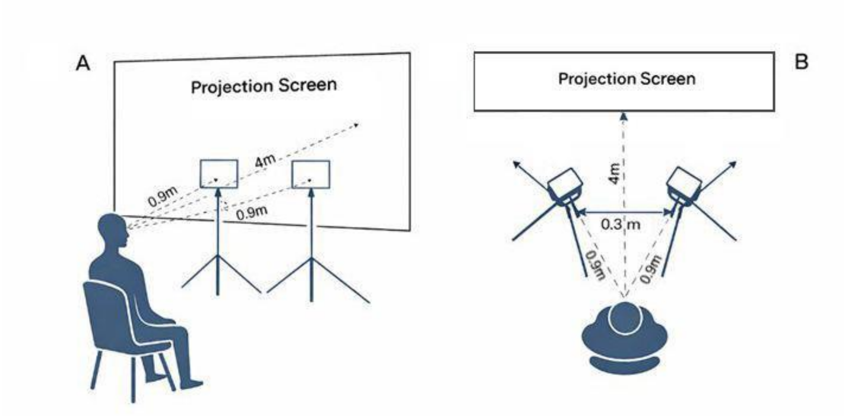

Supplementary Figure S2. Schematic diagram of the experimental light exposure setup. (A) Side view of the participants seated 4 m from the projection wall while viewing the grayscale movie. (B) Top-down view of the two LED light stands positioned approximately 90 cm in front of the participant at eye level, symmetrically angled inward along the line of sight to provide frontal illumination.
